# Co-Use, Simultaneous Use, and Mixing of Cannabis and Tobacco: A Cross-National Comparison of Canada and the US by Cannabis Administration Type

**DOI:** 10.3390/ijerph20054206

**Published:** 2023-02-27

**Authors:** Alanna Chu, Michael Chaiton, Pamela Kaufman, Renee D. Goodwin, Jodie Lin, Chandni Hindocha, Samantha Goodman, David Hammond

**Affiliations:** 1 Ontario Tobacco Research Unit, Toronto, ON M5T 3M7, Canada; 2 Dalla Lana School of Public Health, University of Toronto, Toronto, ON M5T 3M7, Canada; 3 Department of Psychology, University of Ottawa, Ottawa, ON K1N 6N5, Canada; 4 Centre for Addiction and Mental Health, Toronto, ON M6J 1H4, Canada; 5 Mailman School of Public Health, Columbia University, New York, NY 10032, USA; 6 Graduate School of Public Health and Public Policy, The City University of New York, New York, NY 10027, USA; 7 Division of Psychology and Language Sciences, University College London, London WC1H 0AP, UK; 8 School of Public Health, University of Waterloo, Waterloo, ON N2L 3G1, Canada

**Keywords:** cannabis, tobacco, co-use, simultaneous use, mixing

## Abstract

Introduction: Increasing cannabis legalization raises concerns that the use of tobacco, frequently used with cannabis, will also increase. This study investigated the association between the legal status of cannabis in places of residence and the prevalence of cannabis and tobacco co-use, simultaneous use, and mixing by comparing the prevalence among adults in Canada (prior to cannabis legalization) vs. adults in US states that had legalized recreational cannabis vs. US states that had not as of September 2018. Methods: Data were drawn from the 2018 International Cannabis Policy Study, conducted with respondents aged 16–65 in Canada and the US recruited from nonprobability consumer panels. Differences in the prevalence of co-use, simultaneous use, and mixing between tobacco and different cannabis products were examined using logistic regression models by legal status of place of residence among past-12-month cannabis consumers (N = 6744). Results: Co-use and simultaneous use in the past 12 months were most common among respondents in US legal states. Among cannabis consumers, co-use and simultaneous use were less common in US legal states, while mixing was less frequent in US states with both legal and illegal cannabis compared to Canada. Use of edibles was associated with lower odds of all three outcomes, while smoking dried herb or hash was associated with higher odds. Conclusions: The proportion of cannabis consumers who used tobacco was lower in legal jurisdictions despite higher prevalence of cannabis use. Edible use was inversely associated with co-use, suggesting that edible use does not appear to be associated with increased tobacco use.

## 1. Introduction

Cannabis and tobacco are commonly consumed worldwide and are often used together, either concurrently (i.e., co-use of both by the same consumers but on separate occasions), simultaneously (i.e., the simultaneous use of cannabis and tobacco by the same consumers on a single occasion), or by mixing (i.e., co-administration in the form of mulling, spliffs, and blunts) [1]. The heavy burden of morbidity and mortality related to tobacco is well known [2]. However, the use of tobacco with cannabis presents a separate and significant public health concern. This is because a growing segment of cannabis consumers, who may not have otherwise used tobacco, may be exposed to tobacco through co-use, simultaneous use, and mixing.

Multiple studies have found that cannabis consumers are more likely to smoke cigarettes and/or use tobacco in another form relative to those who do not report cannabis use [3,4]. In Canada and the United States (US), 36.3% and 29.1% of cigarette smokers, respectively, also reported cannabis use in the past 12 months [5,6]. One large cohort study found that light tobacco smokers were five times more likely to use cannabis compared to nonsmokers, and that heavy smokers (10+ cigarettes per day) and heavy cannabis consumers (weekly or more) were approximately thirty times more likely to co-use compared to their respective nonconsumers [7]. Moreover, from 2005–2015, nondaily cigarette smoking increased among daily cannabis consumers in the US, despite an overall decline in cigarette smoking [8]. Similarly, the prevalence of daily cannabis use is significantly higher among daily and nondaily cigarette smokers compared to former and never smokers [9] Daily cannabis use increased from 2002–2014 across all smoking frequencies, with the highest rate of increase among former cigarette smokers [9]. Additionally, cannabis use is associated with increased risk of relapse to cigarette use among former smokers [3].

Tobacco and cannabis are used together in what are known as “blunts”, “spliffs”, or “cigarette chasers”. In North America, blunts refer to partially or completely hollowed-out cigar papers or rolled tobacco leaves filled with cannabis [10,11]. Spliffs are loose-leaf tobacco added to cannabis joints, a process known as mulling or mixing, and a cigarette chaser is the use of a tobacco cigarette immediately after a cannabis joint [1,3,10]. Traditionally, studies have found that blunt use is the most common process of combining tobacco and cannabis among cannabis consumers in the US [12], whereas mulling is more common in Europe. In Switzerland, 4 out 5 students who use cannabis add tobacco to their joints [13]. From 2015–2017, 31.1% of adult cannabis consumers in Ontario mixed tobacco and cannabis [14]. This process is also widespread in the United Kingdom and Australia [15,16]. However, the landscape of cannabis use is rapidly evolving. 

Individuals who mix cannabis with tobacco and who would not otherwise use tobacco alone are newly exposed to the negative health effects of tobacco [17]. Additionally, some social cannabis consumers may not be aware that they are also consuming tobacco mixed with their cannabis [17]. Consumers who mix tobacco with cannabis often perceive mixing to be less harmful and addictive compared to using tobacco alone [17]. However, the co-use of both substances has been associated with worse symptoms of cannabis dependence, poorer cannabis cessation outcomes, increased risk of cannabis and tobacco use relapse, lower academic achievement, and higher risk of physical and mental health problems compared to cannabis use only [3,4,18,19,20,21]. Co-use has also been associated with greater respiratory symptoms and the risk of developing mental health problems compared to exclusive use of either tobacco or cannabis [22,23]. Mixing tobacco and cannabis has been shown to increase exposure to carbon monoxide and to result in higher THC inhalation per gram of cannabis [21,24], as well as greater cannabis use-related problems compared to using cannabis exclusively [12]. 

Many countries are moving towards legalization or have legalized nonmedical (herein ‘recreational’) cannabis. On 17 October 2018, recreational cannabis became legal in Canada. To date, recreational cannabis is legal in 18 US states plus the District of Columbia (DC) and medical cannabis is available in 37 states. The public health implications of legalizing cannabis are not yet well understood [25], and importantly, the impact of legalization on rates of co-use and mixing have not yet been examined [26,27]. There are a wide variety of cannabis products available on the market, with various methods of administration. Individual consumers also vary in their frequency of cannabis use. While most cannabis is smoked or vaped as dried herb, there are also oral or vaped oils or liquids, edibles (foods), beverages, solid concentrates, hash or kief, tinctures, and topical ointments. It is believed that most cannabis mixed with tobacco is in the form of dried herb, but little is known about co-use or mixing with other cannabis products. Cannabis legalization also has been shown to diversify the product mix of the cannabis market [28]. 

Most previous research conducted on mixing cannabis and tobacco has been qualitative or descriptive and has not consistently differentiated cannabis and tobacco co-use from mixing. Consequently, it cannot distinguish between the mode of co-use driven by individual characteristics and use driven by the products themselves [17]. Little is known about the patterns of using tobacco and cannabis together across jurisdictions, or associations with the type of cannabis or tobacco products used. A 2017 study found differences between the UK, Canada, and the US in how nicotine was used with cannabis among youth, with higher odds of concurrent use compared to exclusive nicotine use in Canada compared to the US and England [29]. 

This study investigates the differences in prevalence of cannabis and tobacco co-use, simultaneous use, and mixing among adults in Canada (immediately prior to cannabis legalization), US states that had legalized recreational cannabis (‘legal states’) as of September 2018, and those that had not (‘illegal states’). These reflect two different comparisons: one between countries and the second between different stages of legalization. We will examine how three jurisdictions, which differ in terms of their cannabis policy environments, differ in patterns of co-use, mixing, and simultaneous use of tobacco and cannabis. These policy differences may affect type of cannabis use (e.g., dried herb, edibles, oils/liquids) and type of tobacco product use (i.e., cigarette, vape, e-cigarette). The type of product used may also affect how individuals choose to use cannabis and tobacco together (i.e., concurrently, simultaneously, or mixed). We posit that patterns of co-use that reflect the stage of legalization (US illegal --> Canada --> US legal) may be associated with legalization compared with patterns that are more similar between US states compared to Canada that may be associated with historical or cultural differences that are less affected by policy.

## 2. Materials and Methods 

Data are from Wave 1 of the International Cannabis Policy Study (ICPS), conducted in Canada and the US. Data were collected via self-completed web-based surveys conducted from 27 August–7 October 2018, immediately before the legalization of nonmedical cannabis in Canada, with respondents aged 16–65. Respondents were recruited using nonprobability sampling through the Nielsen Consumer Insights Global Panel and their partners’ panels. Email invitations (with a unique link) were sent to a sample of panelists (after targeting for age and country criteria) with cannabis usage unknown; panelists known to be ineligible were not invited. Surveys were conducted in English in the US and English or French in Canada. The cooperation rate, which was calculated based on AAPOR Cooperation Rate #2 as the percentage of respondents who completed the survey of eligible respondents those who accessed the survey link, was 64.2% in 2018 [30]. Median survey time was 19.9 min.

Respondents provided consent prior to completing the survey. Respondents received remuneration in accordance with their panel’s usual incentive structure (e.g., points-based or monetary rewards, chances to win prizes). See Hammond et al. (2020) for more details [5]. The study was reviewed by and received ethics clearance through a University of Waterloo Research Ethics Committee (ORE# 31330). A full description of the study methods can be found in the ICPS: Technical Report—Wave 1 (2018) (www.cannabisproject.ca/methods, accessed on 10 December 2022).

### 2.1. Variables 

All information was self-reported. Cannabis use was defined as having used any cannabis product (i.e., dried herb, edibles, oils/liquids (orally ingested or vaped), hash or kief, concentrates, beverages, tinctures, topicals, or other cannabis products) within the past 12 months. Tobacco use was defined as having used tobacco cigarettes or e-cigarettes/vaped nicotine within the past 12 months. Co-use was defined as reporting both cannabis and tobacco use within the last year. Simultaneous use was defined as having ever used tobacco (i.e., tobacco or e-cigarettes/vaped nicotine) and cannabis on the same occasion in the past 12 months. Respondents who reported using ≥1% tobacco with cannabis dried herb, concentrate, or oil/liquid were coded as having mixed cannabis and tobacco. 

### 2.2. Data Analysis 

A total of 28,471 respondents completed the survey after removing respondents with invalid responses to data quality questions, ineligible country of residence, smartphone use, or residence in DC (due to inadequate sample size) (n = 1302). An analytic sample of 27,169 respondents were included in the current analysis after excluding respondents with missing data, or who responded, ‘Refuse to answer’ or ‘Don’t Know’, and then limiting to cannabis consumers in the past 12 months (n = 6744). 

Post-stratification sample weights were constructed based on the Canadian and US Census estimates. Respondents from Canada were classified into age-by-sex-by-province and education groups. Respondents from the US legal states were classified into age-by-sex-by-legal state, education, and region-by-race groups, while those from the illegal states were classified into age-by-sex, education, and region-by-race groups. Correspondingly grouped population count and proportion estimates were obtained from Statistics Canada and the US Census Bureau. A raking algorithm was applied to the full analytic sample (n = 27,169) to compute weights that were calibrated to these groupings. Weights were rescaled to the sample size for Canada, US illegal states, and US legal states. Estimates are weighted unless otherwise specified. 

Demographic data assessed herein included sex at birth, age group (16–25, 26–35, 36–45, 46–55, 56–65), education (<high school, high school, some postsecondary with no degree, ≥bachelor’s degree), visible minority status (yes/no), and income adequacy (very difficult to very easy). Data for the US were aggregated into legal and illegal states. The prevalence of self-reported cannabis use, tobacco use, tobacco and cannabis co-use, simultaneous use, and mixing were assessed for Canada, US legal, and US illegal states. Prevalence estimates were also stratified by province and state. Overlap between cannabis co-use, simultaneous use, and mixing was also assessed. 

Other variables assessed included sex, age group, education, visible minority status, income adequacy, type of cannabis and tobacco products used, and frequency of cannabis use in each jurisdiction among cannabis consumers. Detailed question wording is available in the ICPS Wave 1 (2018) survey (www.cannabisproject.ca/methods, accessed on 10 December 2022).

Separate logistic regression models were conducted to assess the relationship between type of cannabis product used and co-use; simultaneous use; and mixing among past-12-month cannabis consumers, adjusting for the aforementioned demographic variables. Adjusted odds ratios (aORs) are reported throughout. All analyses were conducted using survey commands in Stata/MP 15.1. 

## 3. Results

### 3.1. Prevalence of Co-Use, Simultaneous Use, and Mixing 

Across the entire sample, the prevalence of cannabis and tobacco co-use within the past 12 months was 14.0% (95%CI: 13.3, 14.6), simultaneous use was 9.9% (95%CI: 9.4, 10.5) and mixing was 5.5% (95%CI: 5.1, 5.9). As shown in Table 1, co-use and simultaneous use were highest among respondents in US legal states, followed by Canada, and US illegal states (*p* < 0.01 comparing Canada and the illegal states). Canada had the highest prevalence of reported mixing, followed by US legal and US illegal states (*p* < 0.01 comparing Canada and illegal states). Table 2 details the proportion of co-use, simultaneous use, and mixing among past-12-month cannabis consumers by jurisdiction. The proportion of cannabis consumers reporting co-use was similar between Canada and illegal states, both of which were higher than US legal states (*p* < 0.01 comparing illegal and legal states). Mixing was higher in Canada compared to illegal and legal states (*p* < 0.01 comparing Canada and the legal states). Other details on prevalence of dried herb mixed with tobacco in these jurisdictions can be found in Goodman et al., 2020 [31]. 

### 3.2. Likelihood of Co-Use, Simultaneous Use, and Mixing 

Compared to Canada, cannabis consumers residing in US legal states had lower odds of co-use and simultaneous use of cannabis and tobacco (Table 3). Respondents in US legal states also had lower odds of co-use and simultaneous use compared to illegal states (*p* = 0.001 and *p* = 0.003, respectively). Those in both US legal and illegal states had lower odds of mixing dried herb with tobacco compared to Canada (*p* = 0.001). There was no difference between legal and illegal states (*p* = 0.582).

The use of dried herb (smoked or vaped), hash or kief, and vaped cannabis oil was associated with higher likelihood of co-use and simultaneous use compared to the other forms of cannabis. Use of oral cannabis oils/liquids, beverages, and hash or kief was associated with mixing. Use of concentrates was also associated with increased odds of simultaneous use, whereas use of topical ointments was associated with lower odds of simultaneous use. Consuming edibles was associated with decreased odds of co-use, simultaneous use, and mixing.

Co-use was lowest amongst those aged 55–65 and highest amongst those aged 36–45. Simultaneous use was lowest among those aged 16–25, while mixing was highest among those aged 16–25. Males were more likely to mix and to co-use, while those with the highest levels of education and income adequacy were less likely to co-use or to use simultaneously, but more likely to mix. There were no differences in co-use, simultaneous use, and mixing by visible minority status.

## 4. Discussion

This study found differences in the patterns of co-use, simultaneous use, and mixing amongst respondents aged 16–65 in Canada and US states that had and had not legalized recreational cannabis. Additionally, there were associations between the type of cannabis or tobacco product and use behaviours. These findings suggest that use behaviours may vary within different policy environments and may have implications for public health messaging and approaches. In particular, the increased use of edibles in legal states may reduce the likelihood of co-use, despite a higher level of cannabis use overall.

The elevated use of tobacco among cannabis consumers has been identified as a significant public health concern, amenable to changes in cannabis public policy [32]. Previous studies have found that co-use prevalence was higher among US legal (or legal medical marijuana) states compared to illegal states [33,34]. This paper is the first to examine the interrelationship of cannabis and tobacco as a multidimensional issue, breaking down this relationship into co-use (the use of both substances by the same user), simultaneous use (the use of both substances by the same user on the same occasion), and mixing (the mixing of the two substances to be ingested together). The findings suggest that while over 70% (according to Table 1) of coconsumers were also simultaneous consumers, mixing represents a less common practice. Other research has found that many mixers do not identify themselves as coconsumers or simultaneous consumers [14]. Efforts and policies that limit mixing and/or inform the public about the potential health risks of mixing (including inadvertently developing nicotine dependence) may help to decrease the associations between cannabis and tobacco use. Currently, Canadian regulations do not permit retail cannabis products to contain nicotine, contain tobacco flavours, or comarket products as per the Cannabis Act and related regulations. Additionally, Lower-Risk Cannabis Use Guidelines suggest consuming cannabis via methods other than smoking and avoiding deep inhalation [35]. Future iterations of these guidelines should additionally recommend that consumers avoid using tobacco in conjunction with cannabis [35].

Consistent patterns of mixing were observed within the US compared to Canada, irrespective of legal status. While the likelihood of co-use and simultaneous use were associated with both legal and illegal status, the fact that mixing was consistently lower in the US suggests the possibility that mixing, as one particular form of simultaneous use, is more reflective of cultural and social practice differences between the countries rather than differences in policies. Mixing also demonstrated a different pattern, with higher levels among younger consumers and those of higher socioeconomic status.

Unsurprisingly, cannabis products that were smoked (dried herb and hash or kief) were associated with each of co-use, simultaneous use, and mixing, while vaping cannabis was associated with co-use and simultaneous use, but not mixing. More surprisingly, the use of oral cannabis oils/liquids and beverages was associated with mixing. Use of edibles was associated with a lower likelihood of co-use, simultaneous use, and mixing. Consumers may be more likely to use edibles as stand-alone products as they are less conducive to mixing with tobacco. Edible use was also higher among women compared to men and among 20–24-year-olds compared to older and younger age groups; similarly, females are less likely to smoke tobacco and cannabis compared to men [36].

A number of theories related to co-use and mixing of tobacco and cannabis have been proposed in the literature. The ‘gateway’ and ‘reverse gateway’ theories propose that tobacco smoking primes consumers to smoke cannabis and vice versa [1]. The ‘common factors’ theory suggests that common factors are responsible for both smoking tobacco and cannabis [37]. The Addiction Vulnerability Hypothesis proposes that individuals have pre-existing neurobiological risk factors that predispose them to tobacco and cannabis use and addiction [24]. Other studies suggest that overlapping economic and environmental factors such as social norms and availability increase the likelihood of initiation and maintenance of both tobacco and cannabis use [3]. Some studies suggest that because cannabis and tobacco are both typically administered via inhalation, cigarette smokers are more likely to continue smoking cannabis past the experimentation stage because of aero-respiratory adaptations resulting from cigarette smoking. This similar route of administration may also serve as a smoking-related cue, where the use of one substance may increase cravings for the other [24]. There is mixed evidence that simultaneous use has a synergistic effect, whereby priming of the endocannabinoid system facilitates the potential for nicotine use [24]. Other studies suggest that one substance may be used to attenuate the withdrawal symptoms of the other, and that quitting both substances produces combined withdrawal symptoms that are more severe than the sum of independent withdrawal symptoms from cannabis and tobacco [24]. Further research is needed to better elucidate the relationship between cannabis and tobacco, and to design public health interventions to prevent these use behaviours.

### 4.1. Limitations

This study is subject to several limitations. Respondents were recruited using nonprobability-based sampling rather than probability-based sampling. The data were weighted by age group, sex, and region in both Canada and the US. However, the study samples were somewhat more highly educated than the national average for the general populations in Canada and the US. In both countries, the ICPS sample had poorer self-reported general health compared to the national population, which is a feature of many nonprobability samples [38], and may be partly due to the use of web surveys, which provide greater perceived anonymity than in-person or telephone-assisted interviews often used in national surveys [39]. The rates of cannabis use were also somewhat higher than the national prevalence; however, this is likely due to the fact that the ICPS sampled individuals aged 16–65, whereas the national surveys included older adults, who may have lower rates of cannabis use. In addition, simultaneous use was assessed by asking respondents to select substances they had used (including tobacco) from a list, and then asking whether they had used the identified substances on the same occasion as cannabis. Literature shows that individuals who smoke tobacco with cannabis may not consider themselves as having used tobacco [14]. Respondents who held these beliefs therefore may not have been identified as simultaneous consumers. Measures of socioeconomic status and racial demographics were limited, and further study is required to understand these relationships in more detail.

### 4.2. Implications

Tobacco and cannabis are substances that are commonly used together and frequently used by the same individuals; however, there are important differences suggesting the potential for regulations to reduce co-use and coadministration of cannabis. Co-use and simultaneous use are more common overall in jurisdictions where recreational cannabis has become legal; yet, among cannabis consumers, rates of co-use were lower in US legal states. One potential explanation is the increase in popularity of edibles in legal jurisdictions, as it appears that the use of edibles is associated with less co-use or mixing of tobacco and may be less likely to promote tobacco use among those who use cannabis compared with other cannabis products. Little is known about why people mix or use cannabis and tobacco simultaneously, and future research is necessary to understand the potential impacts on impairment and cognitive functioning, as well as the effects on dependence. As more jurisdictions legalize the sale of cannabis products, the Lower-Risk Cannabis Use Guidelines should include recommendations not to use cannabis with tobacco.

## Figures and Tables

**Table 1:** Sample demographics and prevalence of co-use, simultaneous use, and mixing.

	Canada		United States—Illegal		United States—Legal	
	n	% [95% CI]	n	% [95% CI]	n	% [95% CI]
**Total Sample**	10,057		9714		7398	
**Sex**						
Female	5845	49.9 [48.7, 51.0]	5968	50.3 [49.1, 51.5]	4887	49.7 [48.2, 51.2]
Male	4212	50.1 [49.0, 51.3]	3746	49.7 [48.5, 50.9]	2511	50.3 [48.8, 51.8]
**Age Groups**						
16–25 years	1325	18.9 [17.9, 20.0]	2209	19.9 [19.1, 20.8]	762	16.2 [15.0, 17.4]
26–35 years	1424	20.4 [19.4, 21.5]	1317	21.4 [20.3, 22.6]	1270	25.5 [24.0, 27.0]
36–45 years	1538	19.3 [18.4, 20.3]	1484	18.9 [17.9, 20.0]	1268	16.9 [15.8, 18.0]
46–55 years	2185	20.9 [20.0, 21.8]	1883	20.1 [19.2, 21.1]	1570	22.1 [21.0, 23.3]
56–65 years	3585	20.5 [19.7, 21.2]	2821	19.6 [18.8, 20.4]	2528	19.4 [18.5, 20.3]
**Education**						
Less than High school	873	14.2 [13.2, 15.2]	1646	15.2 [14.5, 16.0]	358	8.2 [7.3, 9.2]
High school	1548	27.6 [26.4, 28.8]	1567	19.5 [18.5, 20.5]	1003	19.2 [17.9, 20.5]
Vocational training ^1^	4268	33.6 [32.5, 34.6]	2925	38.4 [37.2, 39.7]	2567	46.8 [45.2, 48.3]
≥Bachelor degree	3309	24 [23.1, 24.8]	3551	26.9 [25.9, 27.9]	3456	25.9 [24.9, 27.0]
**Visible Minority**						
No	8864	86.9 [86.0, 87.7]	8604	81.5 [80.3, 82.6]	6610	86.7 [85.6, 87.8]
Yes	1035	11 [10.3, 11.8]	828	14 [13.0, 15.1]	583	9.4 [8.5, 10.4]
Unstated	158	2.1 [1.7, 2.5]	282	4.5 [3.9, 5.2]	205	3.8 [3.2, 4.6]
**Income Adequacy**						
Very difficult	806	8.8 [8.1, 9.5]	847	9.3 [8.6, 10.0]	554	8.4 [7.6, 9.3]
Difficult	2000	21.1 [20.1, 22.1]	2107	22.2 [21.2, 23.2]	1423	20.8 [19.6, 22.0]
Neither easy nor difficult	3593	35.8 [34.6, 36.9]	3016	31.6 [30.4, 32.7]	2443	33.3 [31.9, 34.8]
Easy	2197	20.7 [19.8, 21.6]	2225	22 [21.0, 23.0]	1715	22.4 [21.2, 23.7]
Very easy	1183	10.3 [9.7, 11.0]	1330	12.9 [12.1, 13.7]	1118	12.7 [11.8, 13.7]
Don’t know	131	1.9 [1.5, 2.3]	122	1.4 [1.1, 1.7]	69	1.3 [0.9, 1.7]
Refused	147	1.5 [1.2, 1.8]	67	0.7 [0.5, 0.9]	76	1.1 [0.8, 1.5]
**Past Year Cannabis Use**						
Any	2413	28.3 [27.2, 29.5]	1997	23.8 [22.7, 24.9]	2344	36.8 [35.3, 38.3]
None	7644	71.7 [70.5, 72.8]	7717	76.2 [75.1, 77.3]	5054	63.2 [61.7, 64.7]
Dried Herb (smoked or vaped)	1894	23 [21.9, 24.1]	1641	19.2 [18.2, 20.2]	1760	29.1 [27.7, 30.5]
Cannabis Oils or Liquids—Oral	576	6.4 [5.8, 7.1]	372	4.9 [4.3, 5.5]	580	9.2 [8.3, 10.2]
Cannabis Oils or Liquids—Vaped	459	6.1 [5.4, 6.7]	579	7.2 [6.5, 7.9]	813	14.4 [13.3, 15.6]
Edibles/Foods	1013	11.8 [11.0, 12.7]	727	8.8 [8.1, 9.6]	1278	19.9 [18.7, 21.3]
Beverages	169	2.2 [1.9, 2.7]	137	2.1 [1.7, 2.5]	294	5.8 [5.0, 6.6]
Concentrates (e.g., wax, shatter, budder)	357	5.1 [4.5, 5.7]	259	3.7 [3.2, 4.2]	435	8.6 [7.7, 9.7]
Hash or Kief	537	7.2 [6.6, 8.0]	293	4.1 [3.6, 4.7]	449	9.2 [8.3, 10.3]
Tinctures	147	1.9 [1.6, 2.3]	126	1.6 [1.3, 2.0]	321	5.1 [4.4, 5.9]
Topical Ointments	234	2.7 [2.3, 3.2]	215	2.7 [2.3, 3.1]	555	8.4 [7.6, 9.4]
Other	9	0.2 [0.1, 0.5]	12	0.2 [0.1, 0.3]	4	0.1 [0.0, 0.2]
**Past Year Tobacco Use**						
Any	2377	26.7 [25.6, 27.8]	2321	27.1 [25.9, 28.2]	1571	26.1 [24.8, 27.6]
None	7676	73.3 [72.2, 74.4]	7390	72.9 [71.8, 74.1]	5823	73.9 [72.4, 75.2]
Tobacco Cigarettes	2134	23.6 [22.6, 24.7]	1897	22.2 [21.1, 23.2]	1324	21.7 [20.4, 23.0]
E-Cigarettes/Vaped Nicotine	816	9.9 [9.1, 10.7]	1151	13.9 [13.0, 14.8]	710	13.1 [12.0, 14.2]
**Past Year Co-Use of Cannabis & Tobacco**						
Yes	1154	14.5 [13.6, 15.4]	970	12.4 [11.5, 13.3]	891	16.2 [15.0, 17.4]
No	8899	85.5 [84.6, 86.4]	8741	87.6 [86.7, 88.5]	6503	83.8 [82.6, 85.0]
**Past Year Simultaneous Use of Cannabis & Tobacco**						
Yes	862	10.9 [10.1, 11.7]	686	8.9 [8.1, 9.7]	625	11.6 [10.6, 12.8]
No	9195	89.1 [88.3, 89.9]	9028	91.1 [90.3, 91.9]	6773	88.4 [87.2, 89.4]
**Past Year Mixing of Dried Herb with Tobacco**						
Yes	501	6.3 [5.7, 6.9]	265	3.7 [3.2, 4.3]	284	5.8 [5.0, 6.7]
No	9556	93.7 [93.1, 94.3]	9449	96.3 [95.7, 96.8]	7114	94.2 [93.3, 95.0]

^1^ Some college or technical/vocational training or certificate/diploma, or apprenticeship. NOTE: sex refers to sex at birth.

**Table 2:** Prevalence of co-use, simultaneous use, and mixing among past-year cannabis consumers.

	Canada n = 2413		United States—Illegal n = 1997		United States—Legal n = 2344	
	%	[95% CI]	%	[95% CI]	%	[95% CI]
**Past Year Co-Use of Cannabis & Tobacco**						
Yes	51.2	[48.8, 53.7]	52.1	[49.4, 54.8]	44.0	[41.3, 46.6]
No	48.8	[46.3, 51.2]	47.9	[45.2, 50.6]	56.0	[53.4, 58.7]
**Past Year Simultaneous Use of Cannabis & Tobacco**						
Yes	38.5	[36.1, 40.9]	37.3	[34.7, 40.0]	31.6	[29.1, 34.2]
No	61.5	[59.1, 63.9]	62.7	[60.0, 65.3]	68.4	[65.8, 70.9]
**Past Year Mixing of Dried Herb with Tobacco**						
Yes	22.3	[20.3, 24.4]	15.7	[13.7, 17.9]	15.7	[13.7, 18.0]
No	77.7	[75.6, 79.7]	84.3	[82.1, 86.3]	84.3	[82.0, 86.3]

**Table 3:** Modes of use as independent predictors of co-use, simultaneous use, and mixing among past-year cannabis consumers in Canada and the United States (n = 6744).

	Co-Use	Simultaneous Use	Mixing
	aOR [95% CI]	aOR [95% CI]	aOR [95% CI]
**Jurisdiction**			
Canada (illegal)	ref	ref	ref
US (illegal)	1.1	1.02	0.60 ***
	[0.95, 1.28]	[0.87, 1.19]	[0.49, 0.73]
US (legal)	0.77 ***	0.77 **	0.56 ***
	[0.66, 0.90]	[0.65, 0.91]	[0.45, 0.70]
**Cannabis Use Mode (Yes vs. No)**			
Dried Herb ^1^	1.82 ***	1.96 ***	
(smoked or vaped)	[1.54, 2.15]	[1.62, 2.36]	
Cannabis Oils or Liquids—Oral	1	1.06	1.38 **
	[0.84, 1.18]	[0.89, 1.26]	[1.08, 1.75]
Cannabis Oils or Liquids—Vaped	1.23 **	1.22 *	1.13
	[1.06, 1.44]	[1.03, 1.43]	[0.91, 1.40]
Edibles/Foods	0.80 **	0.73 ***	0.68 ***
	[0.70, 0.92]	[0.63, 0.84]	[0.56, 0.84]
Beverages	1.05	1.02	2.01 ***
	[0.82, 1.34]	[0.79, 1.31]	[1.51, 2.66]
Concentrates	1.19	1.32 *	1.12
(e.g., wax, shatter, budder)	[0.97, 1.46]	[1.07, 1.62]	[0.85, 1.48]
Hash or Kief	1.61 ***	1.70 ***	1.55 ***
	[1.34, 1.93]	[1.41, 2.05]	[1.22, 1.97]
Tinctures	0.79	0.81	0.73
	[0.60, 1.03]	[0.61, 1.08]	[0.51, 1.05]
Topical Ointments	0.88	0.80 *	1.31
	[0.71, 1.07]	[0.64, 1.00]	[0.99, 1.73]
**Age Group**			
16–25	ref	ref	ref
26–35	1.08	1.26 *	0.92
	[0.87, 1.33]	[1.00, 1.59]	[0.69, 1.21]
36–45	1.32 *	1.77 ***	0.93
	[1.06, 1.64]	[1.41, 2.23]	[0.70, 1.23]
46–55	1.15	1.72 ***	0.55 ***
	[0.93, 1.43]	[1.37, 2.16]	[0.41, 0.74]
55–65	0.73 **	1.04	0.33 ***
	[0.60, 0.90]	[0.84, 1.30]	[0.25, 0.45]
**Sex**			
Male	1.20 **	1.11	1.42 ***
	[1.06, 1.36]	[0.97, 1.26]	[1.19, 1.70]
**Education**			
<High school	ref	ref	ref
High school graduate	1.1	1.19	1.21
	[0.86, 1.40]	[0.92, 1.54]	[0.85, 1.73]
Vocational Training ^1^	1.06	1.06	1.53 **
	[0.84, 1.33]	[0.83, 1.35]	[1.11, 2.10]
≥Bachelor’s degree	0.68 **	0.65 **	1.80 ***
	[0.53, 0.86]	[0.51, 0.84]	[1.30, 2.50]
**Visible Minority**			
Yes	ref	ref	ref
No (White)	1.22	0.95	0.94
	[0.97, 1.53]	[0.75, 1.21]	[0.69, 1.27]
**Income Adequacy**			
Very Difficult	ref	ref	ref
Difficult	1.14	1.08	0.86
	[0.90, 1.46]	[0.84, 1.38]	[0.60, 1.23]
Neutral	0.86	0.88	0.97
	[0.68, 1.08]	[0.69, 1.12]	[0.69, 1.37]
Easy	0.84	0.91	0.96
	[0.65, 1.08]	[0.70, 1.18]	[0.67, 1.38]
Very Easy	0.76	0.72 *	1.60 *
	[0.58, 1.00]	[0.54, 0.97]	[1.09, 2.34]

^1^ Cannabis use modes are independent dichotomous variables (Yes/No). By definition, all mixers use dried herb, so this relationship is not assessed. Some college or technical/vocational training or certificate/diploma, or apprenticeship. * Significant tests compared to the reference group *p* < 0.05, ** *p* < 0.01, *** *p* < 0.001.

## Data Availability

Data may be accessed through application. Proposal forms are available at ICPS-CODE-BOOK-2018-Sept-2-2020.pdf (www.cannabisproject.ca, accessed on 10 December 2022).
